# Integrating regional and local monitoring data and assessment tools to evaluate habitat conditions and inform river restoration

**DOI:** 10.1016/j.ecolind.2021.108213

**Published:** 2021-11-01

**Authors:** Francine H. Mejia, Jason M. Connor, Philip R. Kaufmann, Christian E. Torgersen, Eric K. Berntsen, Todd K. Andersen

**Affiliations:** a U.S. Geological Survey, Forest and Rangeland Ecosystem Science Center, Cascadia Field Station, Seattle, WA 98195, USA; b Kalispel Tribe Natural Resources Department, Usk, WA 99180, USA; c U.S. Environmental Protection Agency, Center for Public Health and Environmental Assessment, Pacific Ecological Systems Division, Corvallis, OR 97333, USA; d Department of Fisheries & Wildlife, Oregon State University, Corvallis, OR 97333, USA

**Keywords:** Environmental Monitoring and Assessment, Program, National Rivers and Streams Assessment, Water temperature, Priest River, Salmonids, Incision, Instream habitat

## Abstract

Restoring degraded rivers requires initial assessment of the fluvial landscape to identify stressors and riverine features that can be enhanced. We associated local-scale river habitat data collected using standardized national monitoring tools with modeled regional water temperature and flow data on mid-sized northwest U.S. rivers (30–60 m wide). We grouped these rivers according to quartiles of their modeled mean August water temperature and examined their physical habitat structure and flow. We then used principal components analysis to summarize the variation in several dimensions of physical habitat. We also compared local conditions in the Priest River, a river targeted for restoration of native salmonid habitat in northern Idaho, with those in other rivers of the region to infer potential drivers controlling water temperature. The warmest rivers had physical structure and fluvial characteristics typical of thermally degraded rivers, whereas the coldest rivers had higher mean summer flows and greater channel planform complexity. The Priest River sites had approximately twice as many deep residual pools (*>*50, *>*75, and *>*100 cm) and incision that averaged approximately twice that in the coldest rivers. Percentage fines and natural cover in the Priest were also more typical of the higher-temperature river groups. We found generally low instream cover and low levels of large wood both across the region and within the Priest River. Our approach enabled us to consider the local habitat conditions of a river in the context of other similarly sized rivers in the surrounding region. Understanding this context is important for identifying potential influences on river water temperature within the focal basin and for defining attainable goals for management and restoration of thermal and habitat conditions.

## 1. Introduction

Aquatic ecosystems are under threat globally (Geist and Hawkins, 2016, Gangloff et al., 2016). Freshwater ecosystems, rivers, and streams in particular face enormous demands to support a growing population and a changing climate (Dudgeon et al., 2006, Geist, 2011, Reid et al., 2019). Declines in populations of freshwater fishes in many parts of the world (Mueller et al., 2018, Nicola et al., 2018, Jones et al., 2020, Leidy and Moyle, 2021) and reorganization of fish communities (Comte et al., 2021) highlight the need to develop systematic and integrative approaches that incorporate scale-dependent analyses of monitoring data to identify mechanisms driving these changes (Geist, 2015, Bierschenk et al., 2019, Mueller et al., 2020, Comte et al., 2021, Feio et al., 2021). Restoring degraded rivers and streams requires an evaluation of the fluvial landscape to identify environmental stressors and determine whether landscape features are altered and can be enhanced or restored. However, finding the appropriate scale to assess ecosystems degradation and recovery is difficult because different levels of biological organization recover at different rates (Pander and Geist, 2013).

Much of the research and application of habitat quality monitoring are done at local scales, with some assessments conducted at the regional, national, and continental level (Kamp et al., 2007, Hill and Blair, 2005, Szoszkiewicz et al., 2006, Fernández et al., 2011, Bierschenk et al., 2019, Mueller et al., 2020). In Europe, the Habitats and Water Framework directives require the consideration of modifications to flow regime, sediment transport, river morphology, and lateral channel mobility. As a result, several geomorphological assessment tools have been developed to be integrated with biological monitoring and to evaluate overall ecological conditions (Rinaldi et al., 2017; Belletti et al., 2018; 2017). In the United States, The U.S. Environmental Protection Agency’s (USEPA) Environmental Monitoring and Assessment Program (EMAP) developed a framework in the early 1990s for regional and national scale assessment of aquatic resources to quantify associations between anthropogenic disturbance and the quality of water and habitat (Hill and Blair, 2005).

Field measurements collected using standardized protocols employed by regional and national monitoring programs enable comparisons to be made among rivers that provide a better understanding of their ecological condition. The EMAP and the National Rivers and Streams Assessment (NRSA) field sampling protocols quantify numerous physical habitat attributes organized in several categories: stream size and gradient, substrate size and stability, habitat complexity and cover, riparian vegetation cover and structure, anthropogenic disturbances, and channel-riparian interaction (Kaufmann et al., 1999, U.S. Environmental Protection Agency, 2013). These habitat characterizations were designed to be used as a complement to biological data and other ecological data such as water temperature and land use.

Concerns about declines of cold-water fishes have resulted in extensive water temperature monitoring and restoration expenditures globally (Daigle et al., 2016, Jackson et al., 2016, Isaak et al., 2017). Across the western United States, hundreds of millions of dollars have been spent (Barnas et al., 2015). Combining this water temperature data with regional hydrologic modeling and field assessments of habitat condition can help infer potential processes at play to inform strategies to restore, augment, and conserve salmonid habitat. In the western United States, recent declines in Bull Trout (*Salvelinus confluentus*) and Westslope Cutthroat Trout (*Oncorhynchus clarkii lewisi*) populations have been associated with changes in habitat connectivity, water temperature, channel morphology, and introduction of invasive fish species (Shepard et al., 2005, Al-Chokhachy et al., 2010, Kovach et al., 2017, Howell, 2018), but these studies are mostly in small streams and rivers (*<*30 m wide). Many studies investigating the effects of instream and riparian characteristics on water temperature have also focused on small streams and rivers (Ebersole et al., 2003, Johnson and Wilby, 2015, Ouellet et al., 2017, White et al., 2017, Klaar et al., 2020, but see Jackson et al., 2021, Kalny et al., 2017, Kuhn et al., 2021). Smaller rivers are more vulnerable to heating and cooling than medium- and large-sized rivers due to their lower buffering capacity (Caissie, 2006). Thus, inferences from studies of small streams and rivers may not always apply to medium- and large-sized rivers.

Our objectives were to (1) examine how instream and riparian characteristics relate to thermal conditions in medium-sized rivers (30–60 m wide) across a region, and (2) evaluate how local habitat characteristics measured with standardized survey protocols within a single watershed (Priest River in northern Idaho) can be compared to existing monitoring data from rivers throughout the region. The rivers were grouped based on their mean August water temperature to compare associations with habitat attributes. We hypothesized that the coldest rivers had (1) smaller cross-sectional areas and were steeper because water in these rivers is less exposed to solar radiation and has shorter retention times, (2) higher mean summer flows that increase buffering thermal capacity, and (3) greater channel planform complexity that increases lateral and vertical connectivity and enhances surface water and groundwater exchange. Lastly, by placing our site-specific findings in this regional context, we aim to inform local habitat restoration and management efforts for native salmonids and other coldwater organisms in rivers.

## 2. Methods and materials

### 2.1. Study area

**Fig. 1.**
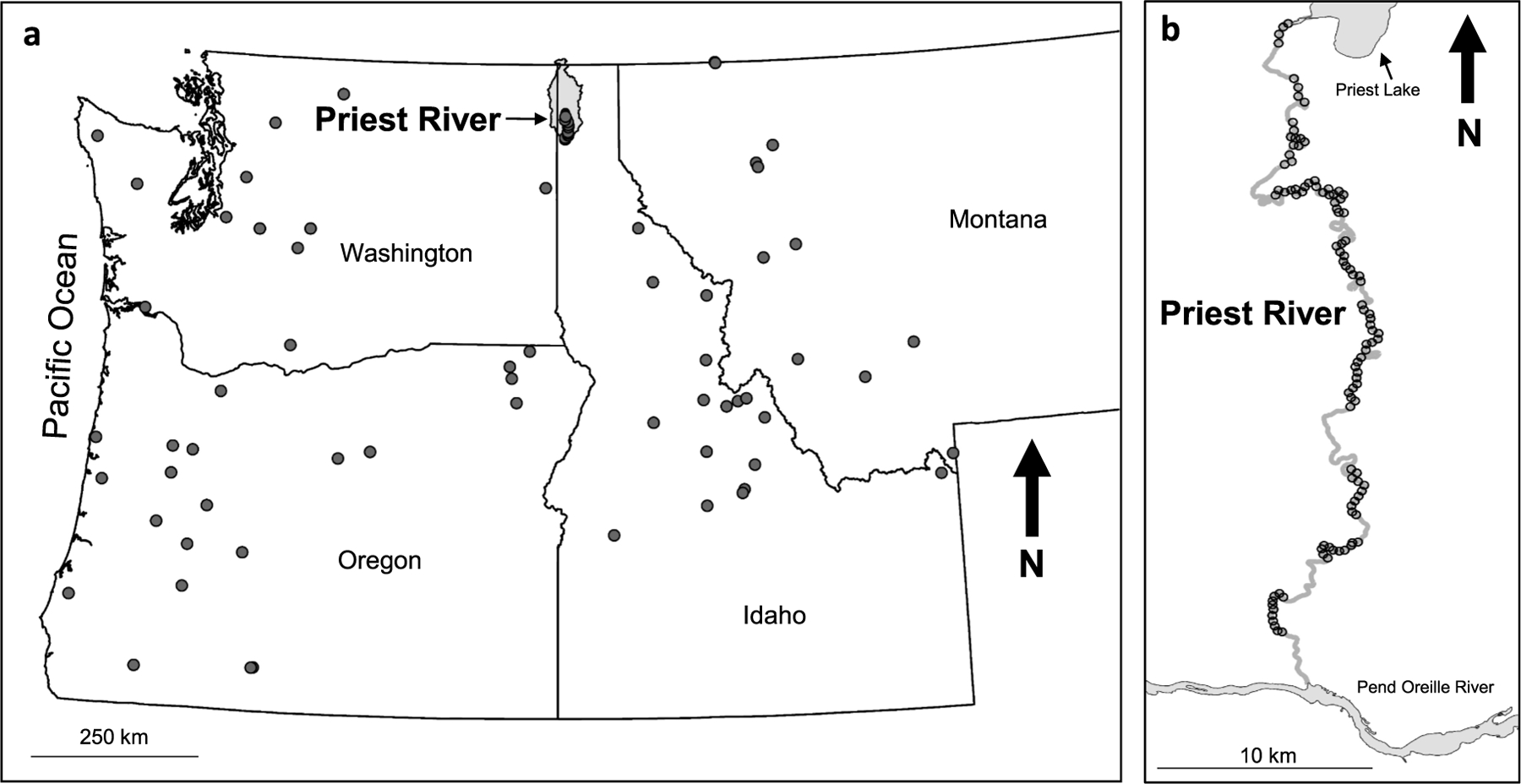
(a) Medium-sized rivers (30–60 m wide) with slopes *<*1% included in the study for comparison with the Priest River sites in northern Idaho, USA (b). All sites were sampled using the U.S. Environmental Protection Agency’s Environmental Monitoring and Assessment Program (EMAP) protocol for non-wadeable streams. Circles represent all sites of medium-sized rivers (a) and transect locations for Priest River (b).

We examined rivers located in the mountains of Idaho, Montana, Washington, and Oregon (USA) that ranged in wetted width from 30 to 60 m, with slopes *<*1% and mean summer flows scaled to drainage area not exceeding 0.06 m^3^s^−1^ km^−2^ (Fig. 1a). These rivers are all within the Marine Coast Forests and Northwestern Forested Mountains Level I ecoregions (McMahon et al., 2001). We refer to this group of rivers collectively as medium-sized rivers. Sites were located along a range of latitudes from 44 to 49° north latitude in Idaho, Montana, Washington, and Oregon. Inland sites were mostly in the Rocky Mountains, whereas the coastal sites were in the Cascade and Pacific Coast ranges. Elevations of sites ranged from close to sea level in the Elochoman River in Washington to over 2,000 m in the Madison River in Montana. Elevation and proximity to the Pacific Ocean drive precipitation in the form of snow or rain that occurs mostly from October to April. We targeted the lower 73 km of the Priest River in northern Idaho for extensive monitoring in 2011 (Fig. 1b) and used NRSA data (2008–2014) for comparison (Fig. 1a).

The lower Priest River is located within the USEPA Northwestern Forested Mountains Level I ecoregion. It originates from the outlet dam at Priest Lake and flows south 73 km to the confluence with Pend Oreille River (Fig. 1b). It has a drainage area of 2,545 km^2^ and elevations ranging from 2,045 m in the Selkirk Mountains to 632 m near the confluence with the Pend Oreille River. Elevations at the sites sampled ranged from 635 m near the confluence to 714 m near the outlet dam. The Priest has a snowmelt-driven flow regime with high flows in late spring (May to June) and early summer and low flows in late summer and winter (August to January). Mean annual flow from 1952 to 2019 at the U.S. Geological Survey (USGS) Gage 12395000 (Priest River near Priest River, Idaho) was approximately 48 m^3^s^−1^ (0.02 m^3^s^−1^ km^−2^). The outlet dam at Priest Lake was first constructed in 1951, but the existing outlet dam was constructed in 1978 (upstream of all our Priest River study sites) to control flows in the summer depending on snowpack and to maintain water levels sufficient for recreational activities and dock access on Priest Lake. The lake is drawn down 1 m in October and flows are unimpeded through the following June.

The geology underlying the mainstem Priest is primarily erodible sedimentary rocks, glacial outwash and till, alluvial deposits, and lacustrian deposits. The surrounding highlands geology consists of an igneous granitic batholith. Accelerated erosion from roads and streambed and bank erosion has led the Idaho Department of Environmental Quality to list the lower 55 km of the Priest from the Upper West Branch Priest River confluence to the Pend Oreille River as impaired for temperature and sediment on the most current (2020) Clean Water Act Section 303 d list, §303(d). The entire mainstem is also impaired for water temperature.

The lower Priest Lake dam is low head and allows upstream passage of salmonids when in spill position. However, native salmonid populations in the lake are depressed due to competition and predation by non-native Lake Trout, *Salvelinus namaycush* (Venard and Scarnecchia, 2005). In the lower Priest River, densities of native salmonids are also low relative to those in nearby rivers (Fredericks et al., 2013). The non-native Smallmouth Bass, *Micropterus dolomieu,* population has been increasing steadily in the mainstem, as are non-native Brook Trout, *Salvelinus fontinalis*, in the tributaries (T. Andersen, unpublished data). Portions of the lower Priest River are used as a seasonal migratory corridor for native adfluvial Bull Trout (DuPont et al., 2007) and Westslope Cutthroat Trout (Andersen, 2016) that migrate from Lake Pend Oreille into the lower Priest River tributaries to spawn.

### 2.2. Sampling protocol and data sources

We paired modeled regional water temperature and flow datasets with field-collected habitat data to assess how the thermal and geomorphic landscape of the mainstem Priest River compares to other similar-sized rivers in the Pacific Northwest and Northern Rockies regions. The EMAP habitat data were collected using a two-stage sampling process for picking a spatially balanced, randomized set of stream monitoring sites (Herlihy et al., 2000). Data were collected at the scale of habitat units at multiple transects and summarized at the reach scale (20 times the channel width upstream and downstream for a total length of 40 times the channel width ranging from 1.2 km to 1.8 km). The reach length was similar to the 1-km resolution of the modeled temperature data. The geographic location of the reach was the midpoint (±50 m to account GPS accuracy). The sampling unit scales for regional and watershed analyses matched the scales of instream and channel processes (Beechie et al., 2010).

Field surveys in the Priest were conducted on August 4–18, 2011 (Fig. 1b) using the EMAP- NRSA protocol for non-wadeable streams (U. S. Environmental Protection Agency, 2013). The sample reach was divided into ten sub-reaches (11 transects per reach) and had a systematic design to locate habitat observations on these stream reaches. The EMAP protocol defines the length of each sampling reach proportionally based on wetted stream width at the time of sampling, with evenly spaced measurements to represent the entire reach. Field crews measured upstream and downstream distances of 20 times the wetted channel width from the predetermined midpoints to center each 40 channel-width field sampling reach.

Survey data were summarized as stream reach-scale attributes describing multiple aspects of riverine physical habitat. These attributes were organized into seven habitat categories: stream size, channel gradient, channel substrate size and type, habitat complexity and cover, riparian vegetation cover and structure, anthropogenic alterations, and channel-riparian interactions (Kaufmann, 1993, Kaufmann et al., 1999). Attributes related to stream size and channel morphology represented riverine habitat quantity (e.g., thalweg depth, depth cross-sections, and wetted and bankfull widths), whereas other attributes provided inferences on the quality of the habitat and potential anthropogenic disturbances. Habitat attributes used in this study are provided in Table 1. Definitions and calculations are described in detail by Kaufmann et al. (1999; 2008) and Faustini and Kaufmann (2007).

We complemented the Priest survey data with data collected in the summer from 2008 to 2014 by the National Rivers and Streams Assessment (NRSA) program to compare the Priest River to similar medium-sized rivers from the USEPA Level I ecoregions (Marine Coast Forests and Northwestern Forested Mountains) (*n* = 75; Fig. 1a). The NRSA is a collaborative program between the USEPA, states, and tribes designed to assess the quality of rivers and streams in USA using a statistical survey approach and consistent field sampling protocols. NRSA data (https://www.epa.gov/national-aquatic-resource-surveys/data-national-aquatic-resource-surveys) includes various measures of human disturbance associated with each site and its watershed (contributing drainage area).

Because of the lack of paired water temperature data for all our sites, we obtained modeled August mean stream temperature data for the 1993 to 2011 period from the NorWeST Interagency Stream Temperature Database and Model, https://www.fs.fed.us/rm/boise/AWAE/projects/NorWeST.html (Isaak et al., 2017). The database consists of regional temperature data (*>*220,000,000 temperature recordings at *>* 22,700 stream and river sites), coupled with spatial statistical network models to develop historical and future climate scenarios at the 1-km resolution (Isaak et al., 2017). Covariates derived from remote sensing, flow gages, and expert consultation were included in the predictive models: elevation, reach slope, percentage of the upstream watershed area composed of lakes, percentage of the upstream watershed area composed of glaciers, annual precipitation, northing coordinate, base-flow index, cumulative drainage area, and percentage riparian canopy coverage, air temperature, discharge and tailwater. Predictive performance of the SSN models is discussed in detail by Isaak et al. (2017).

We also used modeled mean summer flow for the 1915 to 2006 period from the most current Western U.S. Stream Flow Metric Dataset, https://www.sciencebase.gov/catalog/item/508eccefe4b0b59cf7f5a7f8 (Wenger et al., 2010). This dataset contains historical and projected future stream flows for the western USA derived from daily runoff and baseflow predictions from the Variable Infiltration Capacity (VIC) macroscale hydrologic model (Wenger et al., 2010). The VIC model has been employed at regional scales to describe and forecast hydrologic changes. This model has been calibrated and applied mainly to large rivers and has a spatial resolution of 1/8° (latitude–longitude), which is roughly equivalent to a grid of 12 km by 10 km, varying in size depending on latitude (Hamlet et al., 2005). We considered using modeled mean August flow to be consistent with the mean August temperature from the NorWeST Interagency Stream Temperature Database and Model. However, we chose to use modeled mean summer flow instead, because the estimated mean August flow metric predictions are known to be less accurate for sites with strong groundwater influence (Wenger et al., 2010).

### 2.3. Statistical analyses

To calculate physical habitat attributes and condition used in the NRSA program from field surveys in the Priest River, we used the aquamet package for R, version 2.5.1 (Seeliger and Blocksom, 2018). This package estimates over 380 habitat attributes, most of which are subcomponents that contribute to more inclusive summary variables (e.g., numerous wood size class tallies contributing to total wood volume). We used only the summary variables to characterize habitat condition in the Priest River and similarly sized rivers in the region, grouping habitat attributes into four categories: (1) channel size and morphology, (2) substrate size and bed stability, (3) instream and riparian cover, and (4) habitat complexity and riparian human disturbance (Table 1).

To evaluate differences in habitat characteristics along the thermal ranges observed across the region, we calculated the quartiles for the modeled mean August water temperature, including all 85 sites. We grouped the rivers by quartiles but made an additional group for the Priest River sites (Priest). The coldest rivers belonged to the first quartile group (Q1), whereas the warmest rivers belonged to the fourth quartile (Q4) group. The Priest group had two sites in the third quartile (Q3) and 8 sites in the fourth quartile (Q4). We refer to these temperature groups as Q1, Q2, Q3, Q4, and Priest. We then performed a rank-based Kruskal–Wallis test. If the Kruskal–Wallis test was significant (*p <* 0.05), we also performed the post-hoc Dunn test to determine which groups differed from each other.

We used four principal component analyses (PCA) to summarize the reach-scale variation among rivers for each of the habitat condition categories selected. We computed ordinations using centered and unit variance-scaled data for each habitat condition category. We report the variance explained by each principal component (i.e., axis), the loadings for each variable included in the first two axes, and their Pearson correlations. We also created a Pearson correlation coefficient matrix with the resulting eigenvalues of each habitat condition category to describe their relationships. We defined strong correlations as those *>*0.70.

## 3. Results

### 3.1. Characterization of habitat attributes

#### 3.1.1. Channel morphology and stream size

**Fig. 2.**
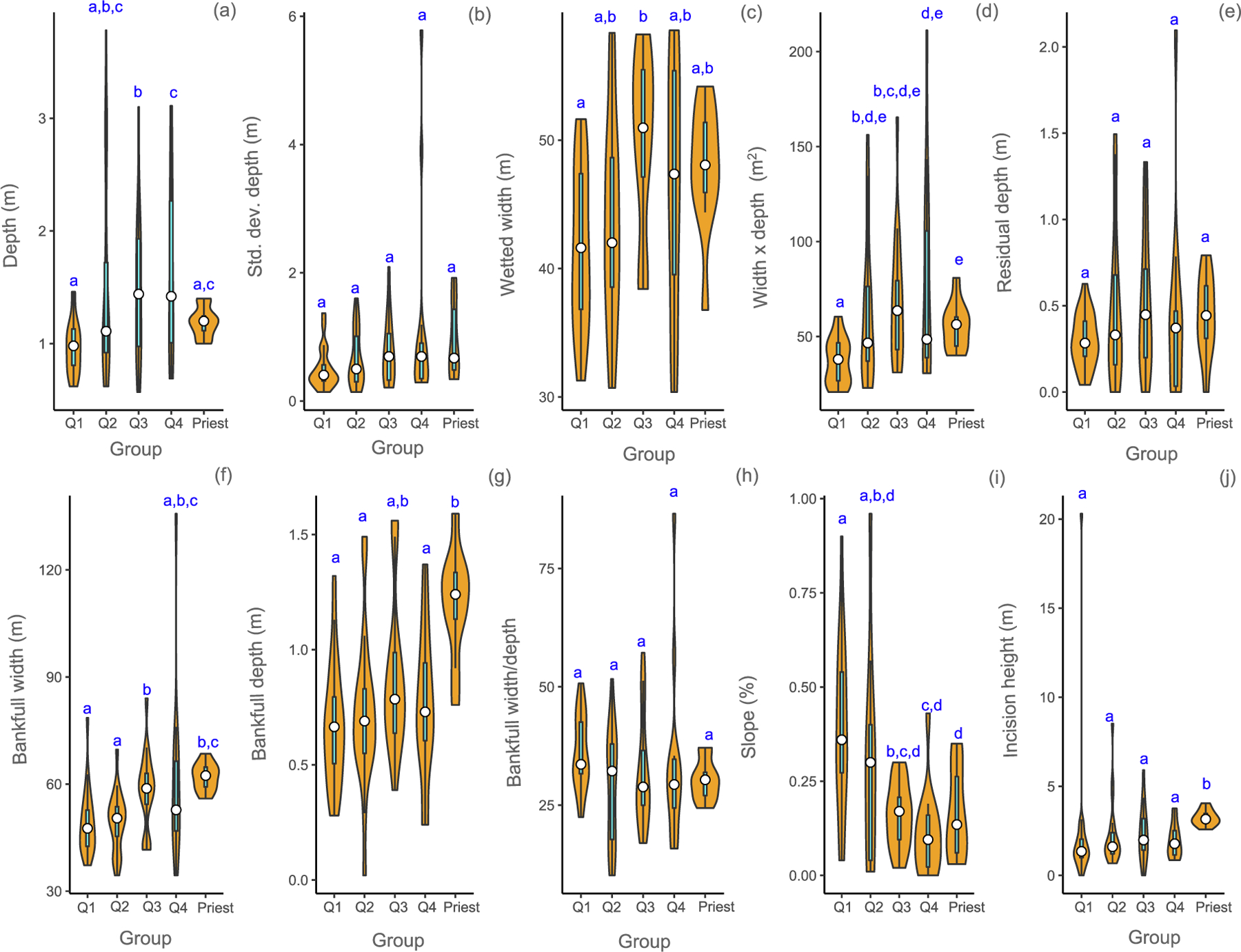
Comparison of channel morphology and stream size attributes (a-j) for mean August temperature groups and the Priest River (Priest) in USEPA Level-I ecoregions (Marine Coast Forests, and Northwestern Forested Mountains). Temperature groups are defined by quartiles (Q1, Q2, Q3, and Q4). Violin plots show the probability density smoothed by a kernel density estimator of each metric by group of the data and include the median (white circle) and the interquartile range (cyan box). Means with different letters indicate a significant difference (Kruskal Wallis test, *p <* 0.05). (For interpretation of the references to color in this figure legend, the reader is referred to the web version of this article.)

The coldest rivers had smaller cross-sectional areas (xwxd) and were steeper (slope) than the warmest rivers (Fig. 2d and Fig. 2i). Mean residual depth (rp100), a flow-independent metric, was not significantly different between warmest and coldest rivers. Although the Priest River had mean residual depth similar to rivers in the Q2 and Q3 temperature groups, it had more deep residual pools than the other groups, with almost twice as many in the *>* 50, *>*75, and *>*100 cm residual depth classes (Table 2).

Mean bankfull width and mean bankfull depth in the Priest River were 62 m and 1.2 m, respectively. The Priest River was approximately 20% wider and almost twice as deep as the coldest rivers (49 m and 0.67 m, respectively). Median incision height was greatest for the Priest River (3.2 m; *p <* 0.02; Fig. 2j) and least for the coldest rivers (1.3 m).

#### 3.1.2. Stream stability and substrate size

**Fig. 3.**
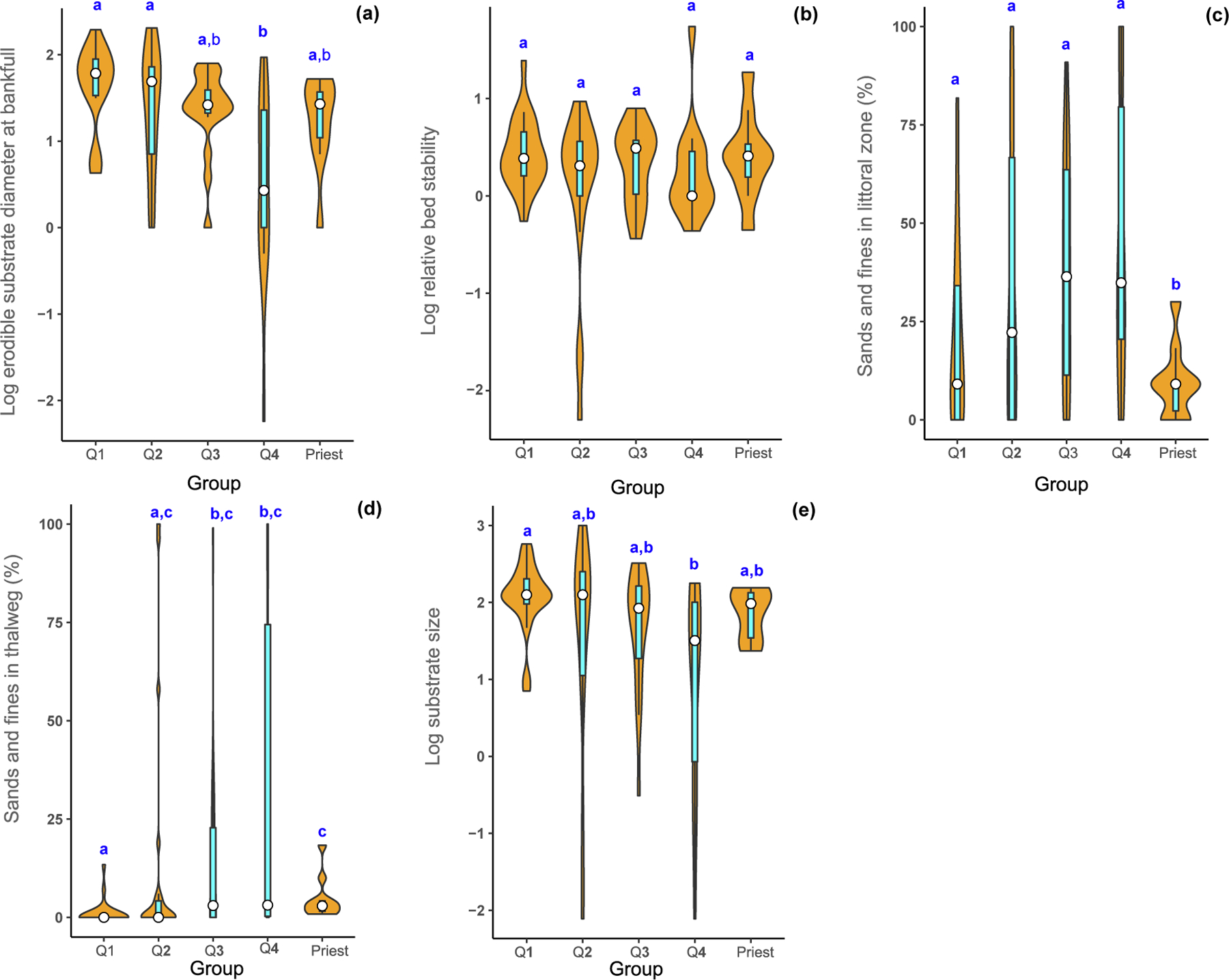
Comparison of stream stability and substrate size attributes (a-e) for mean August temperature groups and the Priest River (Priest) in USEPA Level-I ecoregions (Marine Coast Forests, and Northwestern Forested Mountains). Temperature groups are defined by quartiles (Q1, Q2, Q3, and Q4). Violin plots show the probability density smoothed by a kernel density estimator of each metric by group of the data and include the median (white circle) and the interquartile range (cyan box). Means with different letters indicate a significant difference (Kruskal Wallis test, *p <* 0.05). (For interpretation of the references to colour in this figure legend, the reader is referred to the web version of this article.)

Overall, temperature quartile groups had relatively stable streambeds, but stream bed stability (lrbs_g08) and critical substrate diameter (ldcbf_g08) varied greatly among rivers within their groups (Fig. 3). Substrates were progressively finer from the coldest to the warmest river temperature quartile groups, with substrate in the warmest rivers significantly finer than in the coldest rivers (Fig. 3e). The geometric mean bed surface particle diameter in the thalweg for the coldest group (Q1) and Priest sites was cobble (117 and 72 mm, respectively) whereas the mean substrate size for Q2 and Q3 sites was coarse gravel (37 and 46 mm, respectively). The warmest rivers (Q4) had the smallest mean substrate diameter (10 mm). Mean percentage of sands and fines in the channel thalweg ranged from near 0% in the coldest rivers to 3% in the Priest sites. Sand or finer substrate percentages in the shallower river margins (“littoral” zone) were progressively greater as water temperature increased, except in the Priest River where the average was 9% (median was also 9%).

#### 3.1.3. Instream and riparian cover

**Fig. 4.**
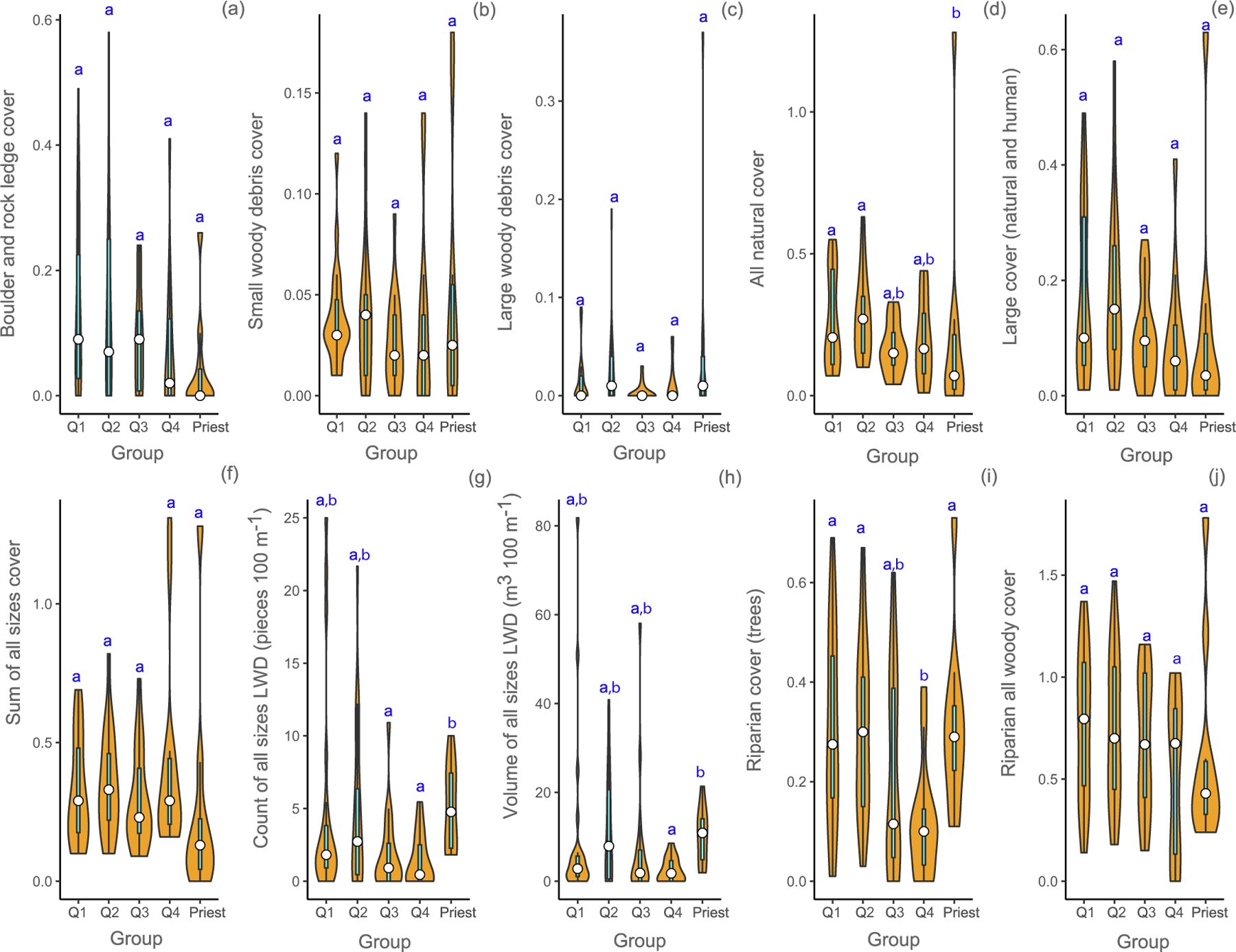
Comparison of instream and riparian cover attributes (a-j) for mean August temperature groups and the Priest River (Priest) in USEPA Level-I ecoregions (Marine Coast Forests, and Northwestern Forested Mountains). Temperature groups are defined by quartiles (Q1, Q2, Q3, and Q4). Violin plots show the probability density smoothed by a kernel density estimator of each metric by group of the data and include the median (white circle) and the interquartile range (cyan box). Means with different letters indicate a significant difference (Kruskal Wallis test, *p <* 0.05). (For interpretation of the references to color in this figure legend, the reader is referred to the web version of this article.)

Attributes related to instream cover were highly variable within the groups. Thus, there were no significant differences between river temperature quartile groups for most attributes except for areal proportions of the sum of all-natural instream cover (Fig. 4d). The mean areal cover proportions for the sum of all-natural instream cover features were *<*0.30 for all groups (Fig. 4d). One site in the Priest River had a proportion *>*1, skewing its mean considerably. Maximum proportions for the other groups did not exceed 0.63 (Q2 group). The mean proportion of the sum of all sizes of areal instream cover ranged from 0.25 to 0.41 (Fig. 4f), but three sites—one in the Priest River and two in the Sprague River (Sprague River belongs to the Q4 group)—had proportions *>*1 influencing the mean values. Mean counts of all size wood were generally low, ranging from 1.4 pieces per 100 m in the warmest rivers to 5 pieces per 100 m in the Priest. Mean volume of large woody debris (LWD) also was lowest in the warmest rivers, approximately one third of the next group, Q3 (2.45 vs. 7.42 m^3^ 100 m^−1^). The mean areal riparian cover proportion from trees of all sizes was low for all groups and ranged from 0.12 in the warmest rivers to 0.31 in the cooler rivers and Priest. The mean riparian woody areal cover proportion (trees, shrubs, and ground) within the groups ranged from 0.53 in the warmest rivers (Q4) to 0.76 in the coldest rivers (Q1).

#### 3.1.4. Habitat complexity and riparian human disturbance

**Fig. 5.**
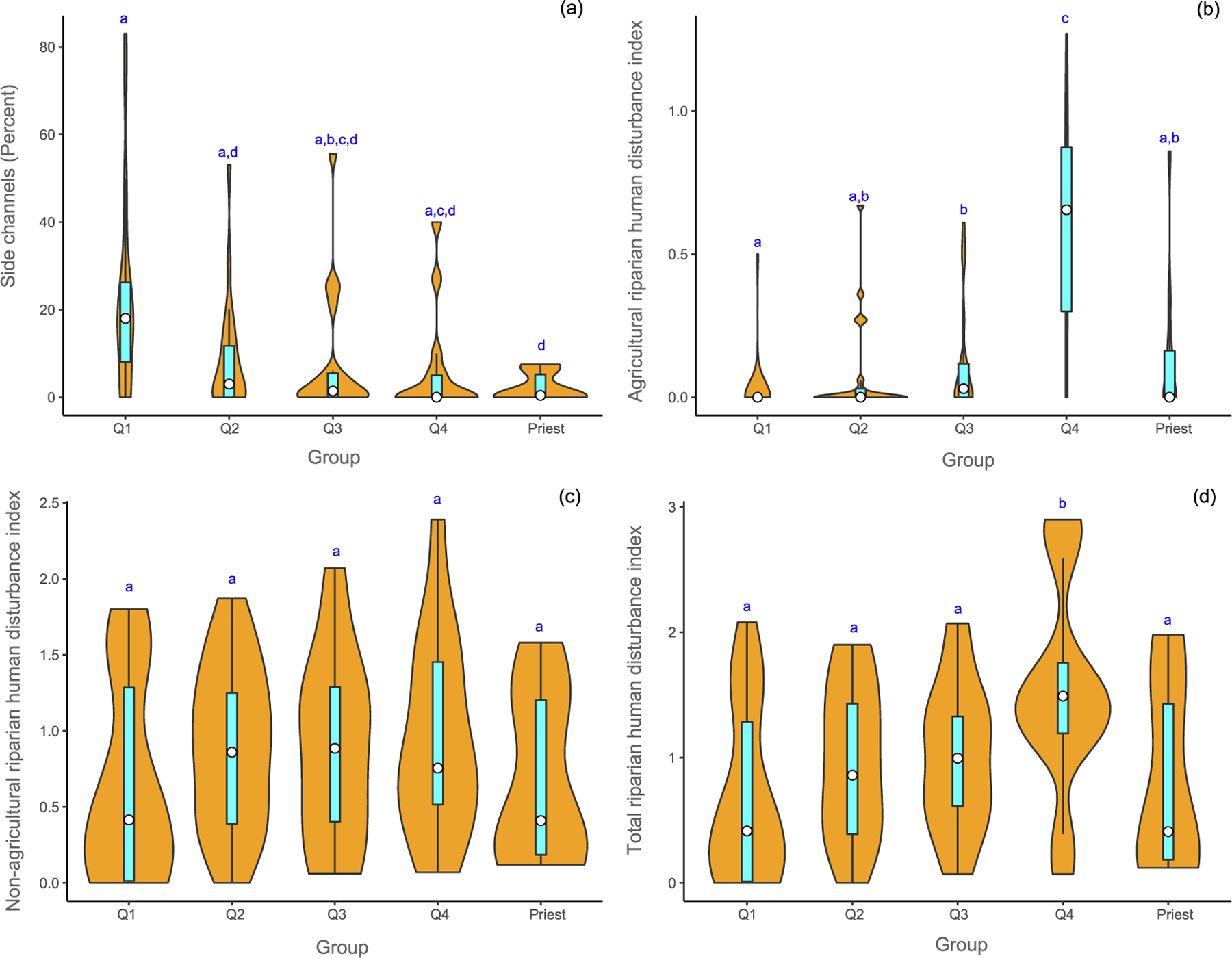
Comparison of indices of percentage of side channels (a), and riparian human disturbance (b - d) attributes for mean August temperature groups and the Priest River (Priest) in USEPA Level-I ecoregions (Marine Coast Forests, and Northwestern Forested Mountains). Temperature groups are defined by quartiles (Q1, Q2, Q3, and Q4). Violin plots show the probability density smoothed by a kernel density estimator of each metric by group of the data and include the median (white circle) and the interquartile range (cyan box). Means with different letters indicate a significant difference (Kruskal Wallis test, *p <* 0.05). (For interpretation of the references to color in this figure legend, the reader is referred to the web version of this article.)

Channel planform complexity, calculated as the percentage of side channels, was lowest in the Priest River (2%, SD ± 3.3), approximately 10% of that in the coldest rivers, which had the highest mean (22%, SD ± 22.4 Fig. 5a). The proximity-weighted indices of total riparian human disturbances were driven by non-agricultural disturbances. The agricultural disturbance index score was about 4% of the total disturbance in the coldest rivers, and 40% in the warmest rivers (Fig. 5b to Fig. 5d). Differences in total riparian disturbances between the warmest and the coldest rivers were significant (*p* = 0.02; Fig. 5d). The warmest rivers had the highest index of disturbance (index of 1.5), whereas the coldest rivers had the lowest (index of 0.67). The mean index of total riparian disturbances in the Priest River was almost 20% more than that of the mean of the coldest rivers (index of 0.82).

### 3.2. Modeled water temperature and areal mean flows

**Fig. 6.**
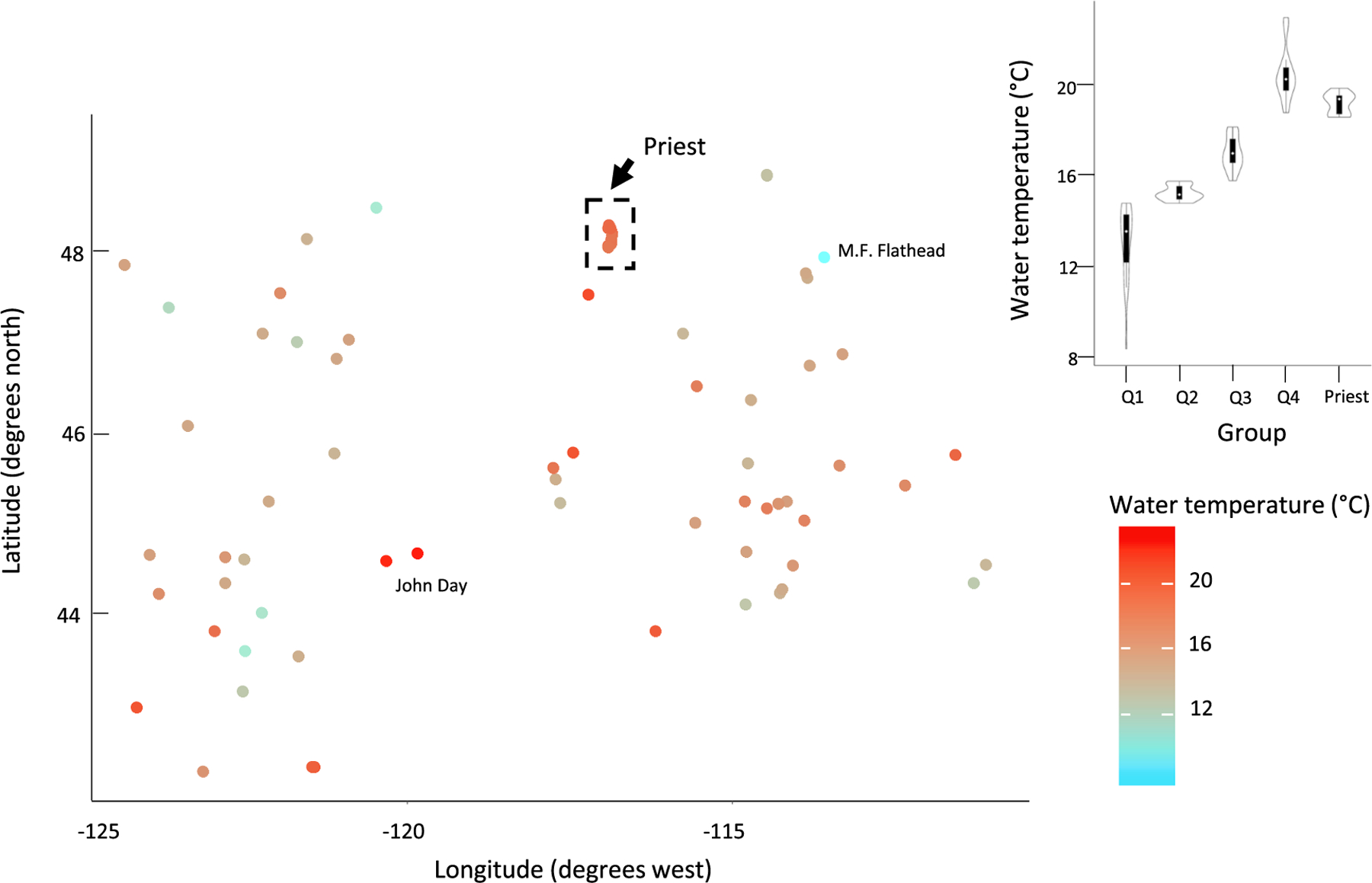
Mean August water temperature for medium-sized rivers in the USEPA Marine Coast Forests and Northwestern Forested Mountains Level I ecoregions. Violin plots show the probability density of each metric by group and include the median (white circle) and the interquartile range (black box).

The Middle Fork of the Flathead River in Montana was the coldest site (8.4 °C), whereas the John Day River in Oregon was the warmest site (23 °C; Fig. 6). The water temperature in the coldest group, Q1, ranged from 8.4 °C to 14.8 °C. In contrast, water temperature in the warmest group, Q4, ranged from 18.8 °C to 23.0 °C. The Priest River water temperatures ranged from 18.6 °C to 19.8 °C.

**Fig. 7.**
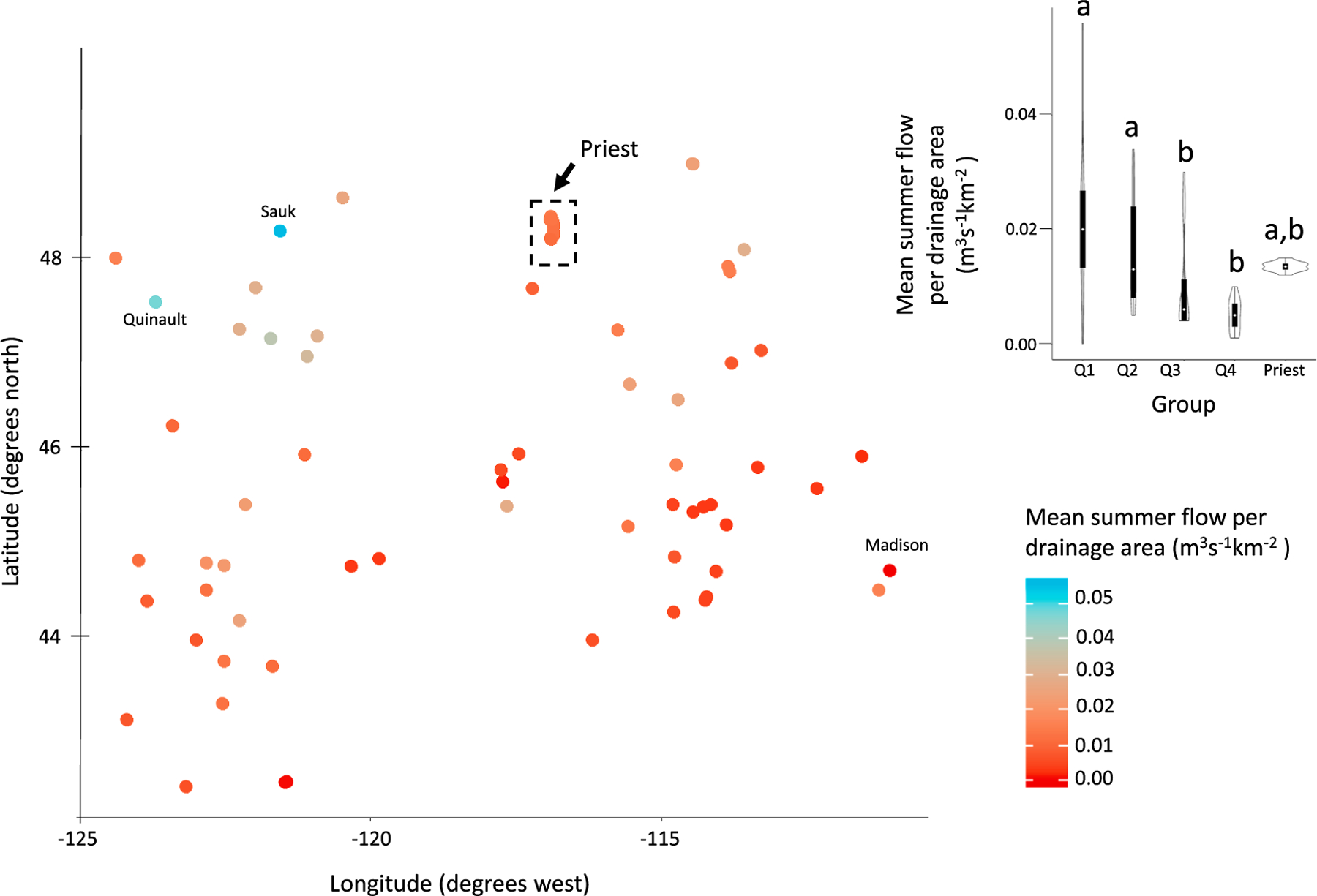
Mean summer flow for medium-sized rivers in the USEPA ecoregions Level I Marine Coast Forests and Northwestern Forested Mountains. Violin plots show the probability density of each metric by group and include the median (white circle) and the interquartile range (black box). Means with different letters indicate a significant difference (Kruskal Wallis test, *p <* 0.05).

Areal mean flows (Fig. 7) were generally lowest in the warmest group (0.005 m^3^s^−1^ km^−2^), whereas flows were over 4 times higher (0.022 m^3^s^−1^ km^−2^) in the coldest group (*p <* 0.0001). The Sauk River, in the Western Cascades, and the Quinault River, on the Olympic Peninsula in Washington, both of which were included in the first quartile group, had the highest summer flows per drainage area (0.056 and 0.048 m^3^s^−1^ km^−2^, respectively). However, the two rivers with the lowest flows per drainage area (0.001 m^3^s^−1^ km^−2^) were the Madison River in Montana (in the coldest group) and the Sprague River in Oregon (in the warmest group).

### 3.3. Variability of habitat conditions

**Fig. 8.**
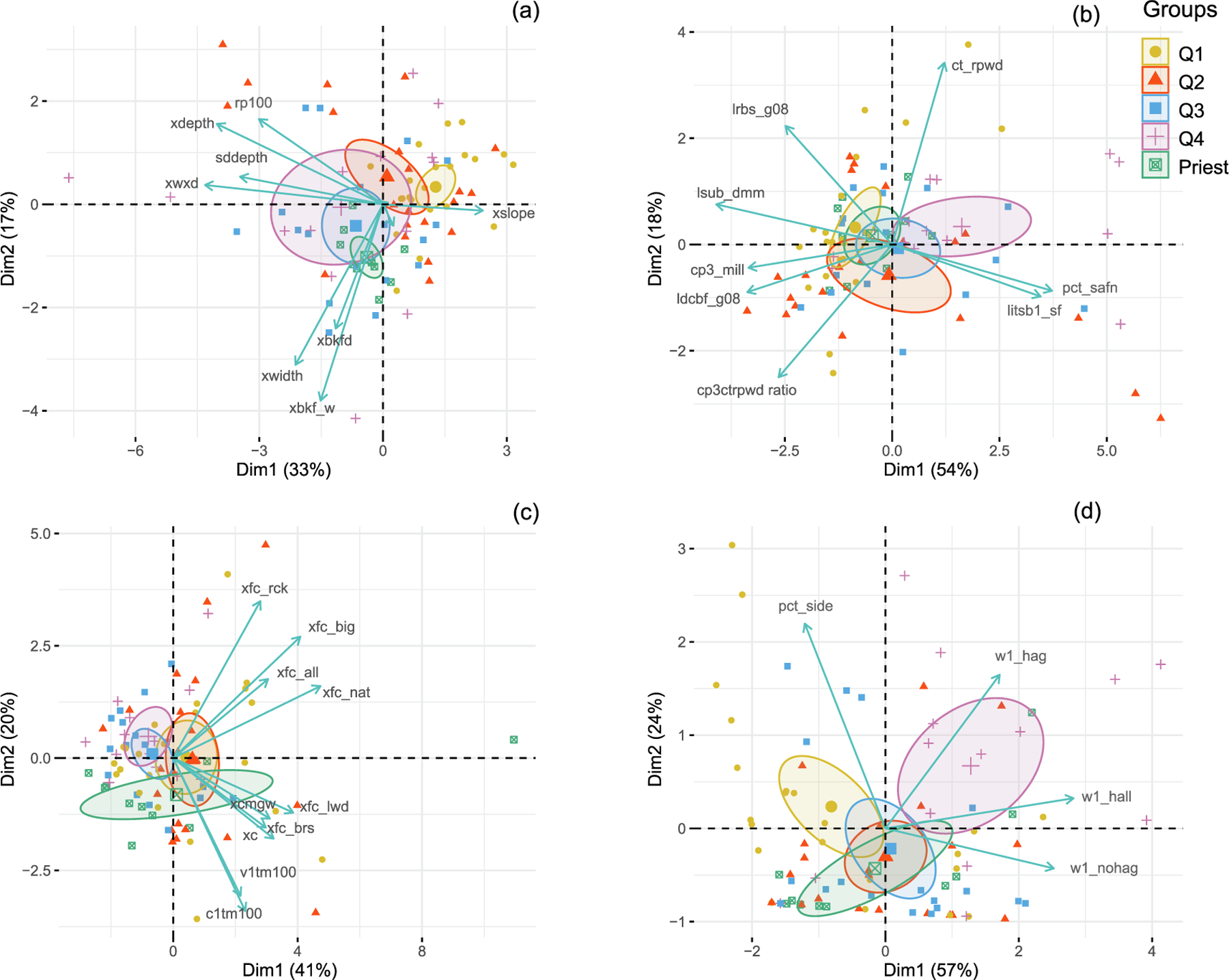
Principal component analysis (PCA) biplots of habitat attributes for (a) channel morphology and stream size (*Morph*), (b) stream stability and substrate size (*Bed*), (c) instream and riparian cover (*Cover*), (d) habitat complexity and riparian human disturbance (*Comp-Dist*) from the NRSA and EMAP surveys conducted from 2008 to 2014 mean August temperature groups and the Priest River (Priest) in USEPA Level-I ecoregions (Marine Coast Forests, and Northwestern Forested Mountains). Temperature groups are defined by quartiles (Q1, Q2, Q3, and Q4). Ellipses represent the 95% confidence intervals on the group means. The biplots explain 50, 72, 61, and 81% of the variance across all sites (*n* = 85) for each of the habitat categories, respectively. Correlations and loadings are reported in Table 3.

To summarize variation in physical habitat among rivers, we computed an ordination of variables in each habitat category (Fig. 8). The first and second axes of the channel morphology and stream size PCA (*Morph*), explained, respectively, 33% and 17% of the variance, with 50% cumulative variance explained. Three out of the 10 habitat attributes, cross-sectional area (xwxd), mean depth (xdepth), and depth variability (sddepth), had strong correlations ≥ |0.70|) with *p <* 0.0001 (Table 3), whereas only two habitat attributes, mean width (xwidth) and mean bankfull width (xbkf_w), had strong (≥ |0.70|) correlations with the second axis. Comparison of the first axis of the *Morph* ordination revealed that the coldest group was significantly different from the other groups (*p <* 0.0001) except for the next warmer group (Q2) and that these differences were mostly driven by slope.

The first two axes of the relative bed stability and substrate size PCA (*Bed*) explained a cumulative variance of 72%, with most of the variability explained by the first axis (54%). For the first axis, five out of the eight habitat attributes had correlations ≥ |0.70|, with *p <* 0.0001; Table 3): substrate size (lsub_dmm), percentage sands and fines in the thalweg (pct_safn), percentage of shallow river margins or littoral zone with sands and fines as dominant substrate (LitSB1_SF), erodible substrate diameter at bankfull or the shear stress of the stream at bankfull flood stage (ldcbf_g08), and bankfull hydraulic resistance from bed particles (cp3_mill). For the second axis, only bankfull total hydraulic resistance (ct_rpwd), had strong correlations. Comparison of the first *Bed* axis revealed that the cooler groups, Q1 and Q2, were significantly different from the warmest group, Q4 (*p* = 0.004, and *p* = 0.03 respectively). These differences were mostly driven by substrate size.

The first two axes of the instream and riparian cover PCA (*Cover*) together explained 61% of the variance. Two-thirds of this variability was explained by the first axis (41%) and only three out of the 10 habitat attributes had significant strong correlations (r ≥ 0.70): sum of all natural areal cover (xfc_nat), sum of all large areal cover (xfc_big), and sum of LWD areal cover (xfc_lwd).

The first two axes of the habitat complexity and riparian human disturbance ordination (*Comp-Dist*) explained a cumulative variance of 81%, the most variability of all four ordinations (Fig. 8). The first axis of the *Comp-Dist* ordination explained 57% of the variance, and the second axis explained 24%. For this ordination, riparian human disturbance index for all types (w1_hall) and riparian human disturbance from other sources than agriculture (w1_hnoag) had strong correlations. Percentage side channels (pct_side) was negatively correlated (*r* = −0.41) with the major axis of human activities, suggesting loss of habitat complexity in response to human activities. Comparison of the first *Comp-Dist* axis revealed that the coldest group, Q1, was significantly different from the warmest group, Q4 (*p* = 0.003). These differences were mostly driven by percentage side channels and riparian human disturbances from agriculture.

We also evaluated the correlation between axes of habitat condition categories. Most of these correlations were not significant (Figure S1). The few significant correlations were weak to moderate and ranged from −0.24 to 0.46.

## 4. Discussion

The results of this study indicate that stream morphology, channel planform complexity and mean summer flows are associated with water temperature in medium-sized rivers through various mechanisms. Steeper rivers with smaller cross-sectional areas are less exposed to solar radiation and have less area for convective heat exchange (Poole and Berman, 2001), whereas rivers with higher mean summer flows and complex channel planforms have greater buffering capacity from thermal inertia and surface water-groundwater exchange (Poole and Berman, 2001, Arrigoni et al., 2008). These results also demonstrate how regional monitoring protocols can be leveraged to evaluate physical habitat and thermal and fluvial conditions at the watershed scale, and then to place these smaller, more detailed basin studies in the context of the surrounding region. We found that the warmest rivers had physical structure (e.g., excess fine sediments, and higher levels of anthropogenic disturbances) and fluvial characteristics typical of thermally altered rivers, whereas the coldest rivers had higher mean summer flows and greater channel planform complexity. The Priest River sites were within the two higher temperature quartiles but shared contrasting habitat characteristics of rivers in both the coldest and warmest temperature groupings. For instance, the Priest River sites had approximately twice as many deep residual pools (*>*50, *>*75, and *>*100 cm) and as much LWD as the coldest sites. In contrast, incision in the Priest River sites averaged approximately twice that in the coldest rivers. Percentage fines, natural cover, and percent of side channels in the Priest River were also more typical of the higher-temperature groups of rivers. We found generally low instream cover and low levels of LWD both across the region and within the Priest River. We compared the Priest River physical habitat with that in other medium-sized rivers subject to a range of thermal conditions within the same ecoregion to understand which processes may be responsible for creating favorable thermal conditions for native salmonids (e.g., lateral, longitudinal, and vertical connectivity). This approach can be used by managers to set baselines, inform decisions and expectations for recovery of salmonids (e.g., determining the extent of restoration or mitigation efforts), and infer potential processes driving water temperature within a basin and region.

The four physical habitat attribute categories described in our study comprise important elements of fish habitat and serve as the template on which aquatic habitat is created (Reid et al., 2020). The quality of this physical habitat, within and above the active channel, and associated processes can be further evaluated to understand how they can limit the distribution of biota, e.g., native salmonids (Geist, 2011). Systematic removal of LWD and boulders, splash damming, and log drives were common across the United States, Canada, and northern European countries like Sweden and Finland (Sedell and Luchessa, 1982, Steel et al., 2017, Tornlund and Ostlund, 2002, Wohl, 2014, White et al., 2017). Scour from these activities and other legacy land uses widened rivers (White et al., 2017) and decreased the frequency of large, deep pools (McIntosh et al., 2000) in the Columbia River basin. In the lower 73 km of the Priest River, log drives occurred from the 1890s to the 1950s and extended almost the entire length of the lower 73 km (Sims, 1998; 2002). Splash dams were not built in the mainstem of the Priest River, and there are records of splash dams in only two tributaries: Big Creek and the lower West Branch Priest River (Sims, 1998). Extensive logging likely increased sediment supply that aggraded channel beds (White et al., 2017) and accelerated deposition on river floodplains (Knighton, 1998). However, excess sediment in the main channel was scoured out as the result of log drives. These two sequential processes, aggradation and incision, led to a greater elevation difference between the floodplain and the channel thalweg. Specifically, the sediment deposited in the floodplain created a greater elevation difference between the floodplain and the scoured channel thalweg, amplifying the degree of floodplain detachment (Wohl, 2004). Our findings on the Priest River are consistent with rivers that have undergone excessive anthropogenic channel scouring. The resultant habitat characteristics include greater mean bankfull depth, greater mean incision height, and low instream areal cover, compared with the least-disturbed streams and historical information (Surian and Rinaldi, 2003, Wohl, 2014). The legacy of log drives, as well as the low head dam that restricts flow from the lake during the summer and releases warm lake surface water, may have led to increased downstream temperatures, simplified geomorphology, sparse instream cover, and decreased amounts of LWD. This corroborates the findings of other studies examining landscape changes affecting biota and channel morphology of rivers and streams (Bierschenk et al., 2019, Poeppl et al., 2015, Wohl, 2015, 2020).

In general, residual pool attributes provide a flow-independent measure of habitat volume and complexity (Kaufmann and Faustini, 2011). Residual pools provide deep water habitat for salmonids during the summer when water temperatures are higher than their optimum temperatures, thereby affecting the carrying capacity of the river (Lisle, 1987). Bull Trout in small streams (*<*10 m wide) are generally absent from reaches where residual pool depths are *<*30 cm (Al-Chokhachy et al., 2010). In our study, the Priest River had two to three times as many deep residual pools (*>*50 cm, *>*75 cm, and *>*100 cm) as the other river groups (Table 2). Some of these pools may stratify thermally, providing refuge to native salmonids thermoregulating in the summer during low flows and when water temperatures are above the optimum for these fish (Mejia et al., 2020). However, the thermal advantage of these large pools to salmonids are severely restricted if these pools are occupied by competing non-native fishes such as Smallmouth Bass and Brook Trout.

Bankfull width and bankfull depth are associated with high flows. Flows that exceed bankfull conditions can access adjacent floodplain habitats important to salmonids because they provide additional rearing capacity during wet months by supporting a more heterogenous landscape with abundant prey, diverse temperatures, low velocities, and refuge from predators (Muhlfeld et al., 2003, Armstrong and Schindler, 2013, Hall et al., 2018). However, extreme bankfull depth may be the result of streambed degradation (incision) and floodplain disconnection where incision prevents high flows from diffusing over the floodplain and causes channel deepening due to very high shear stresses at the bottom of the channel (Schumm et al., 1984, Cluer and Thorne, 2014). The correlations of between axis scores from the ordinations suggest that these mechanisms maybe occurring in the region (i.e., moderate associations between the first axes of *Comp-Dist*, *Morph* and *Bed* ordinations and the second axis of *Cover*). Our results for the Priest sites also suggest that floodplain disconnection may have a warming effect because compared to the coldest rivers in the region, the Priest sites have (1) 100% deeper mean bankfull depth, (2) 2.3 times higher mean incision height, and (3) only 10% of the side channels observed in other rivers.

The narrow range of substrate sizes in the Priest River may be related to the small amount of large-scale channel roughness that potentially provides varied hydraulic conditions to sort cobble, gravel, and fines. Large instream structures (natural or man-made) add hydraulic roughness that also facilitates sediment fining and deposition (Buffington and Montgomery, 1999). Overall, the means for the area of natural cover of fish concealment features were low in all temperature groupings (*<*0.30) but were about a 30 to 40% higher in the coldest rivers (0.28 vs. 0.17). Additionally, mean counts of LWD (1.4–5.0 pieces of all sizes 100 m^−1^) and volume (2.5–11 m^3^ 100 m^− 1^) in rivers throughout the region were low or near the minimum targets set by the U.S. National Marine Fisheries Service (*>*5 pieces 100 m^−1^ with diameter ≥ 1.5 cm and length ≥ 15.2 m. The Oregon Watershed Enhancement Board recommends *>*20 pieces 100 m^−1^ (Fox and Bolton, 2007). Fox and Bolton (2007) found in Washington’s unmanaged forested watersheds that the number and volume of LWD in streams increased with bankfull width, but that these values varied widely from one area to another depending on climatic variations that affected the species composition and size of the riparian trees. They reported the largest counts and volumes in western Washington rivers with bankfull width *>*30 m, where the median counts and volume were 106 pieces 100 m^−1^ and 93 m^3^ 100 m^−1^, respectively. Kaufmann et al. (2008) reported an overall median of 28 pieces 100 m^−1^ (range of 6 to 97) with a median of 7.7 m^3^ 100 m^−1^ (0.99–105) in streams *<*5 m wide in the central Oregon Coast Range. The highest counts in our study (21 pieces 100 m^−1^) were in the Quinault, a Q1 site, but these values were still only 20% of those reported by Fox and Bolton (2007). In contrast, eastern Washington medians were 17 pieces 100 m^−1^ and 7 m^3^ 100 m^−1^ for streams narrower than 30 m (bankfull width) (Fox and Bolton, 2007). Riparian vegetation in the Priest River is more like the forest types found in eastern Washington. However, we found that counts of LWD pieces were about 30% of those reported by Fox and Bolton (2007). The low LWD numbers and volumes in the Priest River are consistent with our understanding of the impacts of scour from log drives.

The mean woody riparian cover from ground layer, mid-layer, and upper layer vegetation ranged from 0.53 (warmest rivers) to 0.77 (coldest rivers), with 25 to 50% coming from large riparian trees *>*5 m tall. This contribution generally decreased as water temperature increased except in the Priest River where the largest contribution was observed (50%). Abundant riparian vegetation supports numerous ecological functions (National Research Council, 2002). For example, the low cover and complexity of riparian vegetation on the Priest River may limit in-channel and floodplain hydraulic resistance, stability of stream banks, interception, evaporation of the incoming precipitation, and buffering of stream temperature. Riparian cover can be described as the relative amount of sky obscured by riparian vegetation at a given point. Shade is influenced by cover but changes throughout each day, as the position of the sun varies spatially and temporally with respect to the canopy cover (Kelley and Krueger, 2005). During the summer, when flows are low and water temperature is high, shade from riparian cover may help to maintain cool downstream temperatures, provided that riparian vegetation is tall enough and the stream is sufficiently narrow (DeWalle, 2008, Jackson et al., 2021).

Given the opportunistic nature of our study (i.e., most data were collected for other monitoring purposes), we were limited with respect to study design. Nonetheless, we were able to make inferences about the region and the Priest River. Most datasets used in this study had been used in previous studies, but they had not been used specifically to analyze medium-sized rivers. Thus, we gained new insights into the relevance of mean summer flows, the small amounts of instream cover, and generally low levels of LWD in medium-sized rivers throughout the region. Additional investigation into the influences of legacy land use and hydrological modifications by dams is needed because it would further inform how past disturbances and current dam-altered flows can affect the magnitude, frequency, duration, timing, and variability of thermal regimes of rivers (Olden and Naiman, 2010). However, this level of analysis was beyond the scope of our study. Understanding past land use in the region may help explain some of the variability observed among groups, considering that current land use may not reflect past land uses (Depauw et al., 2019, Steel et al., 2017). Across the region, further examination of the interaction between epilimnetic releases of warm water from low head dams, reach-scale groundwater-surface water exchange, contributions from cold tributaries, and the effects of water withdrawals is needed to understand how these factors may influence summer thermal conditions. For instance, there were two exceptions in the coldest group where summer flows were low: the Madison River in Montana, and the Wallowa River in Oregon. Both rivers are influenced by groundwater but are affected by irrigation withdrawals that may reduce their summer flows (Clifton et al., 2018, Dwire et al., 2018, Vanderhoof et al., 2019).

## 5. Management implications

The environmental impacts of anthropogenic activities and climate change influence the distribution of native fishes (Bierschenk et al., 2019, Isaak et al., 2015, Mueller et al., 2018, Woodward et al., 2010). Out of the 85 sites included in this study, 62 (73%) have been listed as impaired for water temperature, 22 (26%) for both temperature and sediment, and no sites are listed as impaired for sediment only. Furthermore, most sites in the Q3 and Q4 temperature groups were listed for temperature except for four sites in Idaho: the Payette River (*n* = 2) and the North Fork of the Clearwater River (*n* = 2). The Priest River is listed as impaired for temperature and sediment on the most current (2020) §303(d) list under the Clean Water Act due primarily to temperatures above Bull Trout thresholds and accelerated erosion from unpaved roads and stream bank erosion (https://www.deq.idaho.gov/water-quality/surface-water/total-maximum-daily-loads/priest-river-subbasin/; accessed on April 14, 2021). Excessive sedimentation was not apparent in our results, except in one reach in the shallow river margins or littoral zone (30% of littoral area dominated by *<*2 mm diameter substrates). In the thalweg, percentage sands and fines never exceeded 19% (median of 3%). The median areal percentage of littoral area dominated by sand and silt was 9%. Bryce et al. (2010) estimated that optimum sediment tolerance values (areal percentage of sand and fines) were about 11% and 19% for Bull Trout and Westslope Cutthroat Trout, respectively. In the state of Washington, the percentage of sand and fines is on average 5.5% greater in the river littoral zone or shallow river margins than in the thalweg (Glenn Merritt, Washington State Department of Ecology, unpublished data). Although the data collection approach that we used does not allow a direct comparison, we observed greater areal cover of sand and fines in the shallow river margins, where 9% of the area had *>*50% sand and fines, compared with the thalweg, which had 3% areal cover of sand and fines. These results suggest potential bank erosion and localized deposition due to lower energy in the channel margins and scouring of fines from the thalweg. Consequently, sediment delivered to the channel from bank erosion is evident in the near-bank sediments but is quickly transported downstream and does not remain in the thalweg. However, in one reach where the areal percentage of sand and fines was 18% in the thalweg and 18% of the littoral area, sand and fines were the dominant substrates. In this reach, fine sediment loading was apparently large enough to remain in the thalweg.

In this study, we have elucidated potential processes driving water temperature in the Priest River that are applicable to other medium-sized rivers in temperate regions of the world experiencing similar legacy stressors (Gardeström et al., 2013, Mueller et al., 2018, White et al., 2017). The ecological consequences of channel incision are complex because lowered streambed elevation and disconnection from the floodplain can have multiple effects on lotic and riparian structure and function due to lowered groundwater table, loss of wetlands, lower summer base flows, warmer water temperatures, and loss of riparian plant biomass (Steiger et al., 2005, Cluer and Thorne, 2014, Pollock et al., 2014, Schindler and Smits, 2016). Specifically, incised rivers have greater rates of bank erosion, less sediment deposited onto the floodplains, and lower groundwater and nutrient exchange that collectively may decrease instream and terrestrial floodplain productivity and habitat diversity and affect riparian forest community composition (Pander et al., 2018, Pollock et al., 2014, Schindler and Smits, 2016). These changes to riparian communities also influence stream temperature patterns (National Research Council, 2002).

Although our study did not explicitly determine causal mechanisms, the analyses provide context for process-based restoration in the Priest River and other medium-sized rivers with similar habitat characteristics. Because landscape restoration actions are better than reach-scale actions at improving the health of streams and rivers, integrating these two perspectives better inform the probability of success of local restoration efforts (Palmer et al., 2010). Additionally, at the regional level, the insights we gained on (1) the potential influence of summer flows on mean August temperature, and (2) the small amounts of instream cover and LWD constitute important areas for future research and management.

## 6. Conclusions

In this study, we showed how regional and local habitat monitoring protocols and data combined with streamflow and broad-scale modeled water temperature data can be integrated to determine baseline conditions that inform and guide riverine restoration and management efforts across a large region. We compared habitat conditions from the Priest River to other rivers throughout the Pacific Northwest region of the USA and, specifically, to rivers representing a range of thermal conditions. These findings underscore the need to investigate how historical land use may have contributed to the thermal degradation of rivers. For example, the small amounts of instream cover and LWD in rivers across the region suggest that riparian vegetation may have decreased due to land-use changes. Further investigation of these patterns with historical aerial photography and data on past forestry practices is needed to better understand the temporal context of habitat change in rivers throughout the region.

## Supplementary Material

Sup 1

## Figures and Tables

**Table 1:** Distributions of selected reach-scale habitat attributes to describe multiple aspects of riverine physical habitat in the Priest River (Idaho, USA). Sites were sampled on August 4–18, 2011. These attributes were organized into categories: hydrologic variables, channel morphology and size, stream stability and substrate size, instream and riparian cover and large woody debris (LWD), and index of riparian human disturbance. The same habitat attributes were measured or estimated at 75 sites based on the NorWeST mean August water temperature quartiles (Q1, Q2, Q3, and Q4). Citations: (1) Isaak et al., 2017; (2) Wenger et al., 2010; (3) Kaufmann et al., 1999; (4) Kaufmann et al., 2008; (5) U.S. Environmental Protection Agency, 2013.

Habitat metric	Definition	Citation	Units	Median	Median	SD	Min	Max
*Hydrologic variables*								
S1_93_11	Mean August water temperature (NorWeST 1993–2011)	1	°C	19.4	19.2	0.47	18.6	19.9
MS_FcmsDA	Mean summer flow (VIC output 1915 to 2006) scaled to drainage area	2	m^3^s^−1^ km^−2^	0.014	0.014	0.001	0.012	0.015
*Channel morphology and size*								
xdepth	Mean thalweg depth	3	cm	120	120	13	100	140
sddepth	Standard deviation of thalweg depth	3	m	0.67	0.96	0.60	0.34	1.92
xwidth	Mean wetted width	3	m	48.1	47.8	5.0	36.8	54.2
xwxd	Mean wetted width × depth	3	m^2^	56.4	55.6	12.5	40.0	80.9
rp100	Mean residual depth	3	[cm; equivalent to residual pool vertical profile area(m^2^ 100 m^−1^ of reach)]	44.4	44.0	23.7	0.0	79.2
xbkf_w	Mean bankfull width	3	m	62.4	62.0	3.8	55.9	68.5
xbkf_dc	Mean bankfull depth	3	m	1.2	1.2	0.2	0.8	1.6
xbfwd_ratc	Bankfull width to depth ratio	3	m m^−1^	50. 5	53.8	13.8	37.1	84.8
xslope	Water surface gradient for the reach	3	%	0.13	0.16	0.12	0.03	0.35
xinc_h	Mean incision height	3	m	3.2	3.2	0.4	2.6	4.1
*Streambed particle size and stability*								
cp3_mill	Bankfull hydraulic resistance from bed particles	4	dimensionless	0.01	0.006	0.005	0.0	0.01
ct_rpwd	Bankfull total hydraulic resistance including that from large-scale features (wood, pools, etc)	4	dimensionless	0.02	0.02	0.02	0.0	0.06
cp3ctrpwd	Ratio of cp3_mill and ct_rpwd	4	dimensionless	0.28	0.33	0.25	0.0	0.74
ldcbf_g08	Log10[Erodible (Critical) substrate diameter at thalweg during flow bankfull]	4	mm	1.43	1.23	0.53	0.0	1.72
lrbs_g08	Log10[Relative bed stability] at thalweg	4	dimensioness ratio: Log (Dgm Dcbf^−1^)	0.41	0.41	0.45	− 0.35	1.27
LitSB1_SF	Percentage of littoral (shallow river margins) observations sites that have sand or fines as the dominant substrate.	5	%	9.1	9.4	9.2	0.0	30
pct_safn	Thalweg substrate percentage sand + fines (<2 mm)	3	%	2.9	4.8	5.5	0.9	18.3
lsub_dmm	Log10[estimated geometric mean thalweg substrate diameter]	3	mm	1.99	1.86	0.32	1.37	2.19
*Cover (instream, LWD and riparian)*								
xfc_rck	Boulder and rock ledge areal cover	3	Areal cover proportion	0.00	0.04	0.08	0.00	0.26
xfc_brs	Brush and small woody debris areal cover	3	Areal cover proportion	0.03	0.05	0.07	0.00	0.18
xfc_lwd	Large woody debris areal cover	3	Areal cover proportion	0.01	0.05	0.11	0.00	0.37
xfc_nat	Sum of cover from large wood, brush, overhanging vegetation, boulders and undercut banks	3	Areal cover proportion	0.07	0.21	0.39	0.00	1.28
xfc_big	Sum of cover from large wood, boulders, over-hanging banks and humanstructures	3	Areal cover proportion	0.03	0.11	0.19	0.00	0.63
xfc_all	Sum of areal cover from all fish concealment types except algae and aquatic macrophytes	3	Areal cover proportion	0.13	0.25	0.38	0.00	1.28
xc	Riparian canopy cover	3	Areal cover proportion	0.29	0.31	0.18	0.11	0.73
xcmgw	Riparian woody cover, sum of 3 layers	3	Areal cover proportion	0.43	0.62	0.49	0.24	1.78
c1tm100	Large woody debris in and above active channel	3	Pieces 100^−1^ m	4.77	5.05	3.01	1.82	10.00
v1tm100	Large wood debris volume in and above active channel	3	m^3^100^−1^ m	10.87	10.25	6.35	1.90	21.35
*Habitat complexity and riparian human disturbance*								
w1_hag	Riparian human disturbance index - Agricultural types	3	Proximity-weighted sum	0.00	0.15	0.27	0.00	0.86
w1_hnoag	Riparian human disturbance index - Non-agricultural types	3	Proximity-weighted sum	0.41	0.67	0.57	0.12	1.58
w1_hall	Riparian human disturbance index - All types	3	Proximity-weighted sum	0.41	0.82	0.78	0.12	1.98
pct_side	Percentage side channel	5	Channel length (%)	0.42	2.25	3.27	0.00	7.50

**Table 2:** Number of residual pools of various depths per reach for sites across the study area (mean and range). Columns represent groups based on the NorWeST (Isaak et al., 2017) modeled mean August water temperature quartiles (Q1, Q2, Q3, and Q4) and Priest (Priest River).

Number of residual pools per reach	Q1	Q2	Q3	Q4	Priest
Residual depth *>* 50 cm	5 (0–8)	4 (0–8)	5 (0–8)	3 (0–6)	9 (0–12)
Residual depth *>* 75 cm	3 (0–6)	3 (0–7)	3 (0–7)	2 (0–5)	6 (0–9)
Residual depth *>* 100 cm	2 (0–6)	2 (0–6)	3 (0–7)	2 (0–5)	4 (0–9)

**Table 3:** Principal component analysis (PCA) correlations and loadings for all rivers and all habitat attributes with variables arranged in order of strength of association (Pearson correlation and loading) with axis 1 and variance (%) and cumulative variance (%) for axis 1 and axis 2. Four different ordinations are listed with their associated variables and loadings: *Morph, Bed, Cover, and Comp-Dist*. Pearson correlation coefficients in bold are *>*0.70 and are considered strong correlations.

Variable	Axis 1		Axis 2	

	*r*	loading	*r*	loading
PCA-Morph: Channel morphology & size				
xdepth	−**0.87**	0.76	0.34	0.11
sddepth	−**0.75**	0.56	0.12	0.01
xwidth	−0.46	0.21	−0.67	0.45
xwxd	−**0.93**	0.87	0.08	0.01
rp100	−0.65	0.42	0.36	0.13
xbkf_w	−0.33	0.11	**−0.82**	0.67
xbkf_dc	−0.25	0.06	−0.52	0.27
xbfwd_ratc	0.03	0.00	0.01	0.00
xslope	0.52	0.27	−0.03	0.00
xinc_h	0.05	0.00	−0.09	0.01
Variance explained (%)	33		17	
Cumulative variance explained (%)	33		**50**	
PCA-Bed: Stream stability & substrate size				
lsub_dmm	**−0.95**	−0.46	0.18	0.15
pct_safn	**0.87**	0.42	−0.20	−0.17
LitSB1_SF	**0.80**	0.39	−0.23	−0.19
ldcbf_g08	**−0.79**	−0.38	−0.21	−0.18
cp3_mill	**−0.78**	−0.37	−0.10	−0.09
cp3ctrpwd	−0.62	−0.30	−0.58	−0.49
lrbs_g08	−0.58	−0.28	0.52	0.44
ct_rpwd	0.28	0.14	**0.80**	0.67
Variance explained (%)	54		18	
Cumulative variance explained (%)	54		**72**	
PCA-Cover: Instream & riparian cover				
xfc_nat	**0.91**	0.45	0.31	0.22
xfc_big	**0.78**	0.39	0.52	0.36
xfc_lwd	**0.74**	0.37	−0.23	−0.16
xc	0.62	0.31	−0.34	−0.24
xcmgw	0.60	0.30	−0.26	−0.18
xfc_all	0.59	0.29	0.34	0.24
xfc_brs	0.57	0.28	− 0.30	− 0.21
xfc_rck	0.54	0.27	0.67	0.47
c1tm100	0.44	0.22	−0.65	−0.46
v1tm100	0.42	0.21	−0.59	−0.41
Variance explained (%)	41		20	
Cumulative variance explained (%)	41		**61**	
PCA-Comp-Dist: Habitat complexity and riparian human disturbance				
w1_hall	**0.98**	0.65	0.11	0.12
w1_hnoag	**0.88**	0.58	−0.15	−0.15
w1_hag	0.60	0.40	0.57	0.59
pct_side	−0.42	−0.28	**0.77**	0.78
Variance explained (%)	57		24	
Cumulative variance explained (%)	57		**81**	
