# Supplementary material for: Integrating regional and local monitoring data and assessment tools to evaluate habitat conditions and inform river restoration
Source: Ecol Indic. Author manuscript; Available in PMC 2022 Nov 1. (PMC8597654; doi:10.1016/j.ecolind.2021.108213)

**Figure S1.** Pearson correlation coefficient matrix of the first and second ordination axes of each habitat category. Significant correlations *(p* < 0.05) are bold and underlined. Habitat categories in principal components analysis (PCA): Comp-Dist = Habitat complexity and riparian human disturbance; Cover = Instream, LWD, and riparian cover; Bed = Streambed particle size and stability; Morph = Channel morphology and size.


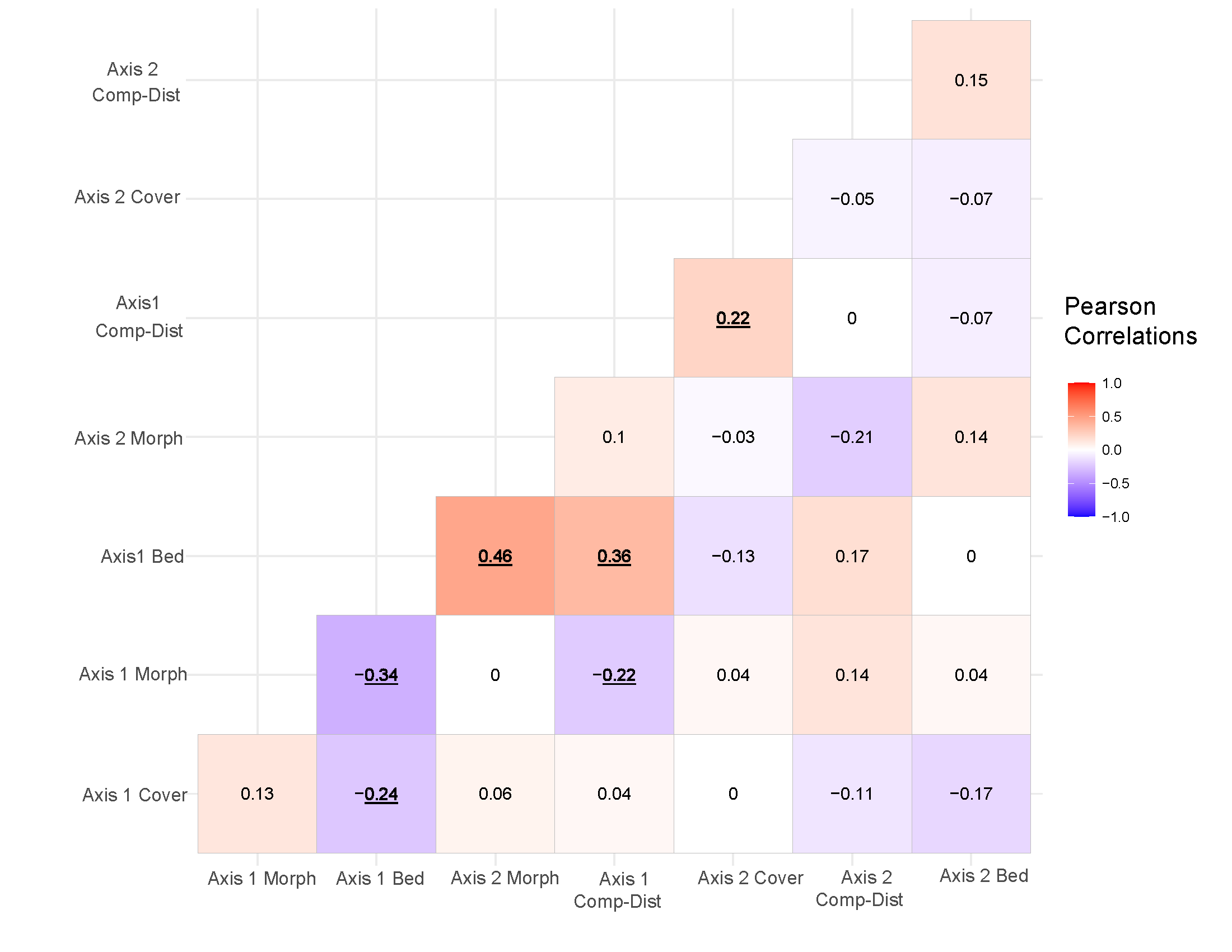

Supplement: Sup 1 [file NIHMS1742503-supplement-Sup_1.docx]
